# Clearing the fog: Australian medical students and the e-cigarette knowledge void – a cross-sectional survey

**DOI:** 10.1186/s12909-025-08126-2

**Published:** 2025-11-17

**Authors:** Samiksha Mali, Vimbiso Chiodze, Hubertus Jersmann

**Affiliations:** 1 Bachelor of Medicine and Bachelor of Surgery (MBBS), University of Adelaide, Adelaide, Australia; 2 Discipline of Medicine, Royal Australasian College of Physicians (FRACP), University of Adelaide, Adelaide, Australia

**Keywords:** E-cigarette, electronic cigarette, medical education, medical curriculum, curriculum development, vaping, knowledge, medical students, smoking cessation, Australia

## Abstract

**Background:**

E-cigarettes (vapes) are increasingly popular, particularly among youth, and pose health risks such as nicotine addiction and lung damage. Despite this, medical students often lack adequate education on vaping. Given their future role in healthcare, it is crucial for them to be well informed. This study examines the attitudes, knowledge, and confidence of Australian medical students regarding e-cigarettes and their education on the topic.

**Methods:**

A cross-sectional survey was conducted among clinical medical students at the University of Adelaide (*n* = 550) to assess their knowledge of e-cigarettes, their attitudes towards vaping, and their confidence in their ability to discuss the subject with patients. The survey, which was open for 3 weeks, included 35 multiple-choice questions covering demographics, vaping knowledge, attitudes, and educational preferences. The data was analysed via descriptive statistics and inferential tests.

**Results:**

Of the 103 initial respondents, 80 completed the survey. The results revealed a significant knowledge gap: 98.75% of the students reported no formal education on vaping, and 97.5% felt that their education was inadequate. Despite this, students scored an average of 5.2 out of 8 on vaping-related knowledge questions. The average self-reported confidence score for discussing vaping with patients was low (3.24/12), with female students reporting slightly greater confidence than males did. Vaping-related information primarily came from social media (58%) and personal networks (41%). A majority of the students (63.75%) preferred vaping education to be introduced during the preclinical years, with small group tutorials being the preferred teaching method.

**Conclusion:**

Australian medical students face significant gaps in vaping knowledge and confidence, which could impair their ability to counsel patients effectively. These findings highlight the urgent need for targeted vaping education in medical curricula in a country that has been otherwise progressive in its nicotine related reforms. Addressing these gaps will better equip future healthcare professionals to address the growing public health concern posed by e-cigarettes.

**Supplementary Information:**

The online version contains supplementary material available at 10.1186/s12909-025-08126-2.

## Background

E-cigarettes, commonly known as vapes, are electronic devices that deliver nicotine-containing aerosols to users by heating a liquid composed of nicotine, vegetable glycerine, propylene glycol, flavouring, and, at times, harmful chemicals such as acetone and herbicide [1]. Initially, designed to help cigarette smokers quit, the marketing of vapes is increasingly targeting nicotine-naive youth, placing them at risk of addiction and future cigarette use [2]. In 2022–23, 57.6% of Australians aged 18–24 years who had ever vaped were completely naive to cigarettes prior to their first vape [3]. A 2022 report from the Australian Department of Health demonstrated that nonsmokers who vape are three times more likely to begin regular smoking than those who do not vape [4]. In Australia, a 2023 national survey revealed that nearly half of the respondents aged 15–30 reported current or past use of e-cigarettes [5]. Another study reported a fourfold increase in vaping prevalence among high school students over the past five years [6]. Alarmingly, 60% of vape cartridges labelled “nicotine-free” do contain nicotine, with most devices delivering amounts equivalent to approximately 50 cigarettes [7]. Nicotine is highly addictive and can impair memory, learning, and mood, particularly in adolescents, in whom exposure may cause lasting structural and functional brain changes [8].

Since 2022, the Thoracic Society of Australia and New Zealand (TSANZ) has warned that e-cigarettes expose users to harmful chemicals and potential carcinogens, leading to both acute and chronic health risks, including lung injury, cancer, and poor oral health [9]. E-cigarette-related poisonings have also increased significantly; calls to Australian poison information centres more than doubled between 2020 and 2021, with most cases involving toddlers as well as adults [10]. Proliferating reports of burn injuries from vape battery explosions have prompted the Australian National University’s (ANU) National Centre for Epidemiology and Public Health to conclude that e-cigarettes can be blamed for serious burns and, in some cases, death [11].

In May 2023, the Australian Federal Government announced reforms banning all retail sales of e-cigarette products, with pharmacy-only access beginning 1 July 2024 [12, 13]. This study was conducted in 2024, amid significant regulatory shifts in Australia’s approach to e-cigarettes. While access was initially proposed as prescription-only, over-the-counter sales were ultimately permitted in most states and territories [14]. The Pharmaceutical Society of Australia has since collaborated with the government to develop training guidelines for pharmacists to support these reforms [13, 14]. Concurrently, TSANZ reaffirmed its support for prescription-only access, noting that no e-cigarette product can be independently recommended as safe or effective for smoking cessation [9]. According to national reviews, including the National Health and Medical Research Council (NHMRC) and ANU reports, there is low to very low certainty of evidence comparing nicotine e-cigarette use to no intervention or usual care. While short-term e-cigarette use may help some smokers who have not succeeded with other aids, most continue to smoke, and safer, proven cessation alternatives are available [10, 15]. In 2024, the Therapeutic Goods Administration (TGA) implemented stricter controls on flavour, nicotine concentrations, and product availability, with restrictions on age and importation practices. Further amendments under TGO 110, which took effect from 1 March 2025, will restrict product names, labels that attract a younger audience, and set further limits on nicotine, menthol flavouring, and container volumes [16].

In this time of flux, this project was motivated by the significant role that medical students may play in shaping societal views on vaping. As young individuals and future healthcare professionals, they are uniquely positioned to interact with peers who use e-cigarettes, and their education provides them with credibility in discussions. In Australia, 32% of 18–24-year-olds—potential peers of medical students—have used vapes [3], whereas 40.5% have used vapes in New Zealand [17]. Despite increasing legal restrictions, exposure remains common [18]. Therefore, future healthcare professionals must be equipped with accurate knowledge about the clinical, pharmacological, and regulatory aspects of e-cigarettes to counsel patients effectively.

Despite the importance of this topic, few studies have examined the attitudes and knowledge of medical students regarding vaping, and none have focused on Australian cohorts. Among the six studies identified, two were from Saudi Arabia [19, 20], one from Spain [21], and the rest from the United States [22–24]. A notable percentage of students across these studies reported engaging in vaping despite being aware of its dangers, with prevalence rates ranging from 4.8% to 37.5%, which aligns with trends observed in Australian and New Zealand university populations [3, 17]. Many medical students believe that vapes pose lower risks for diseases such as cancer than cigarettes do, which is consistent with the opinion of the general user. However, this does not imply that medical students consider vapes harmless and they do recognize negative health consequences. Overall, up to 94.8% of the students perceived their education on vaping to be inadequate, which impacted their confidence in counselling patients [19–24]. Following students, healthcare professionals also reported uncertainty about e-cigarettes [25]. While generally not recommending e-cigarettes, some practitioners, including some cardiothoracic surgeons, support their use in cases involving persistent smokers with related comorbidities or if patients are perioperative [25, 26]. Among alcohol and drug service providers, only a minority believe that e-cigarettes help smokers quit or that they are safer than smoking [27].

The primary objective of this survey was to explore the confidence levels of medical students at our institution in discussing vaping with patients and to investigate the associations among their knowledge of vapes, linked adverse effects, and related laws. We also aimed to examine the sources of information that medical students rely on, both within and outside their medical education, and to assess whether factors such as year level or personal use of tobacco products influence their knowledge.

Given the rapid changes in social and political attitudes towards vaping, we hypothesized that many students may lack confidence in assessing or counselling individuals who use e-cigarettes.

Two of the authors, both recent graduates, received limited formal instruction on vaping during their medical training. In contrast, traditional cigarette smoking was covered in greater depth through lectures, clinical workshops, and online modules. The curriculum addressed the pathophysiology of smoking-related lung inflammation, as well as public health measures targeting tobacco use and smoking cessation counselling. These topics were often reinforced through Objective Structured Clinical Examination (OSCE)-style assessments. Vaping, however, was not present in any of these educational components. While no Australian studies have evaluated the outcomes of such education on smoking cessation, a Chinese study reported that 85% of students felt confident enough to offer smoking cessation counselling [28].

Owing to the lack of structured education about e-cigarettes, we hypothesized that the year level would have a negligible effect on students’ knowledge. Furthermore, while we expected knowledge of vaping legislation to be limited among students, they may still be informed about potential harm through individual research, news sources, or social media.

## Methods

### Participants and setting

The study targeted clinical medical students in their fourth, penultimate, and final years at the University of Adelaide (*n* = 550). All the students received a link to the online survey via their university emails and notifications on Canvas, the university’s learning management system. The survey was open from July 14, 2024, to August 4, 2024. Students were encouraged to participate through a reminder email sent two weeks after the survey began. Additionally, the survey link was shared in Facebook groups exclusive to University of Adelaide students, and reminders were sent the day before the survey closed to increase participation among non-responders. Students were also approached in person to raise awareness about the survey and its deadlines. The study received ethics approval from the University of Adelaide Ethics Committee (Approval No. H-2023-277).

### Measures

The survey items for the questionnaire were loosely adapted from 2 previous studies: a survey of medical students in Minnesota and a multinational survey of dental students [22, 29]. The full details of the questionnaire are available in an additional file (Study Questionnaire). It was administered anonymously via SurveyMonkey, with participants’ IP addresses used solely to prevent duplicate responses. All the participants were asked to answer 35 multiple-choice questions designed to take less than 10 min to maximize participation.

The questions were organized into five categories:

- Demographics: Year, sex, and current vaping habits.
- Knowledge: Understanding e-cigarette contents, legislation, and popularity in Australia.
- Attitudes: Students’ perceptions regarding e-cigarette use.
- Confidence: Self-assessment of confidence and capabilities in discussing e-cigarettes.
- Education: Suggestions for integrating e-cigarette education into the medical curriculum.

The participants were not required to complete every question, but those who answered less than 50% of the survey were excluded from the final cohort.

### Analyses

Data management was conducted via password-protected Excel sheets shared via the university Dropbox among the principal investigators. The primary outcomes included students’ personal experiences with e-cigarettes, knowledge regarding the topic, confidence in their ability to discuss e-cigarettes with patients, and suggestions for improving their education.

The secondary analyses explored how other variables—such as gender, year in medical school, and personal use of vapes—affect students’ knowledge and confidence. The dependent variables included correct vs. incorrect responses in the knowledge section (assessed through four-option multiple-choice or true/false questions), agreement levels regarding attitudes, and preferences for education methods.

For the knowledge questions, the responses were converted to binary data: correct vs. incorrect. A single-factor ANOVA was used to compare knowledge across the three-year groups. Confidence was quantified by treating responses (“agree,” “disagree,” or “neither”) as numerical data, resulting in a confidence “score” for each student. The attitude questions utilized a three-point Likert scale and were analysed via logistic regression. For categorical questions regarding preferred learning methods, a chi-square test was performed. Paired t tests were used to compare responses regarding personal experiences and the impact of gender on confidence. P values less than 0.05 were considered statistically significant. Only complete surveys were analysed, and data analysis was performed via Excel with the Analysis ToolPak add-in.

## Results

### Participants

Out of an estimated cohort of 550 clinical-year medical students, 103 initially responded to the survey. One student did not consent to participate, and 22 left the survey incomplete, resulting in a final analysis cohort of 80 students. Among these participants, approximately 62.5% were female (*n* = 50).

The responses were distributed across the three-year groups, with the youngest cohort being the most responsive, comprising 37.5% (*n* = 30) of the total responses, followed by Year 6 (*n* = 29) and Year 5 (*n* = 21).

With respect to tobacco use, 58.75% of the participants reported having tried a tobacco product, including shisha, cigarettes, or vapes (*n* = 47). Six students identified as current users; among them, five used vapes (83.3%). Notably, all six current users had family members who vaped, contributing to a total of 57.5% of the students reporting that they had family members who used vapes.

### Primary outcomes

**Fig. 1:**
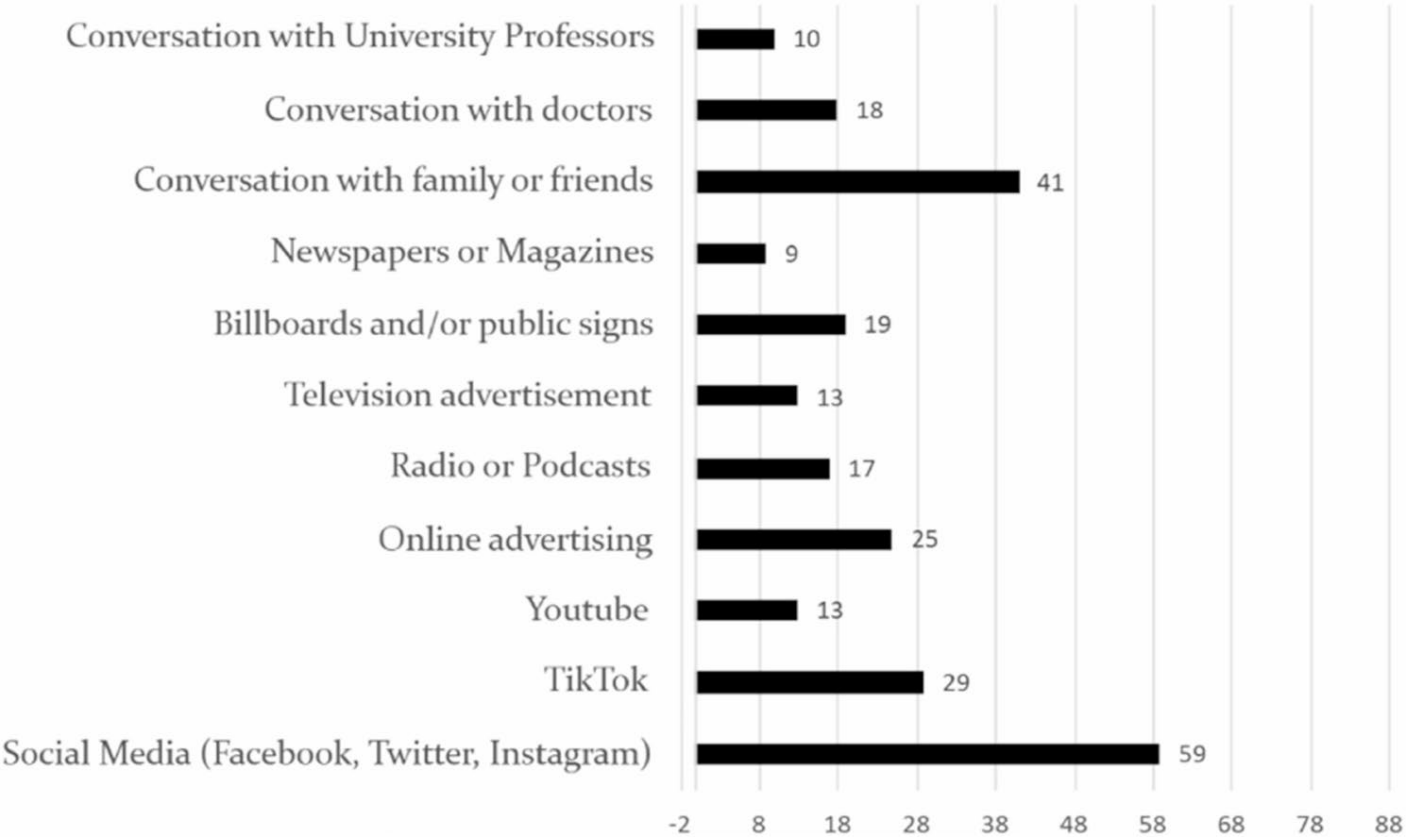
Sources of information regarding e-cigarettes amongst medical students

Regarding formal education on vaping through university curricula, 98.75% of the students reported that they had not received any education on this topic in medical school (*n* = 79/80). Similarly, 97.5% felt that the education provided was inadequate (*n* = 78/80). The two most common sources of information outside of medical schools were social media platforms such as Facebook, Twitter, and Instagram (58/80) and conversations with family and friends (41/80) (Fig. 1). Only 10 students reported gaining information through discussions with university professors.

**Fig. 2:**
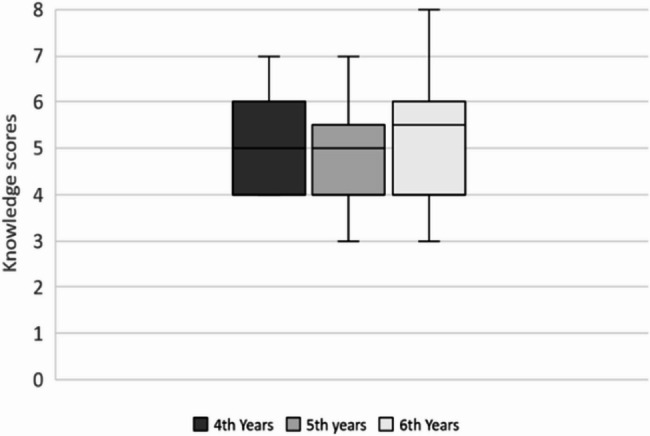
Knowledge scored compared between year 4–6 medical students

Despite the lack of formal education, students scored an average of 5.2 out of 8 on the knowledge section, with a mode of 5. We did not find a statistically significant relationship between knowledge scores and the year level of the participants (Fig. 2).

When asked where in the curriculum, further education about vaping would be best suited, only 1 student chose an optional/selective course. Most students (63.75%, *n* = 51/80) preferred to receive this education during their theoretically led preclinical years.

**Fig. 3:**
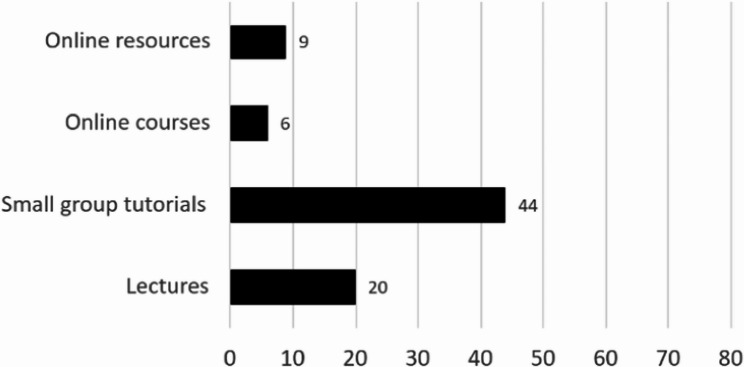
Preferred learning methods regarding e-cigarettes

There was a statistically significant difference in preferences for how vaping education should be delivered (x^2^ = 44.65, *p* < 0.001), with small group tutorials being the most favoured teaching method (*n* = 43/80) (Fig. 3).

### Secondary outcomes

The average self-reported confidence score for students discussing vapes was 3.24 out of a possible 12-point scale, with the most common score being 0. There was a statistically significant difference between the average confidence scores of male and female medical students (t = 2.352, *p* < 0.01). The female medical students reported a higher average confidence score of 3.9, whereas the average confidence score for the male students was 2.06.

In contrast, there was no statistically significant difference between the knowledge scores of males and females. Compared with male students, female students had a slightly higher average knowledge score (5.3) (5.03).

Thirty-eight of the 80 students were comfortable discussing vapes with family members, whereas only 14 were comfortable discussing them with patients. Intriguingly, those who cited conversations with family or friends as a source of their knowledge had better knowledge scores (t = 2.001, *p* < 0.05).

**Fig. 4:**
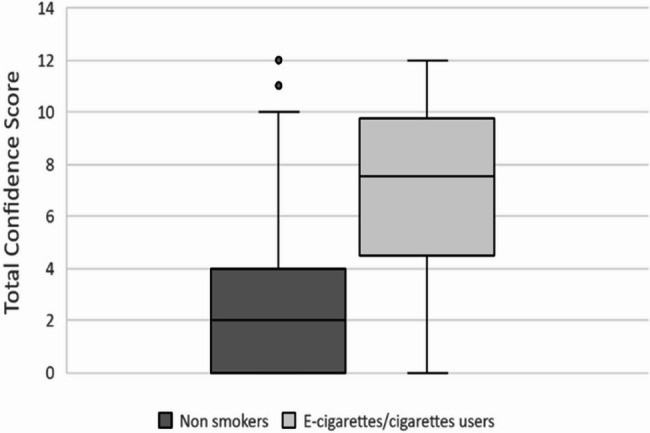
Knowledge scored compared between nonsmokers and e-cigarettes/cigarette users

Students who were current smokers or vapers reported higher confidence scores on average than those who were not using tobacco or vaping products (t= -3.822, *p* < 0.001). However, we did not find a significant difference between vape users’ knowledge scores and non-vape users’ knowledge scores (Fig. 4).

**Fig. 5:**
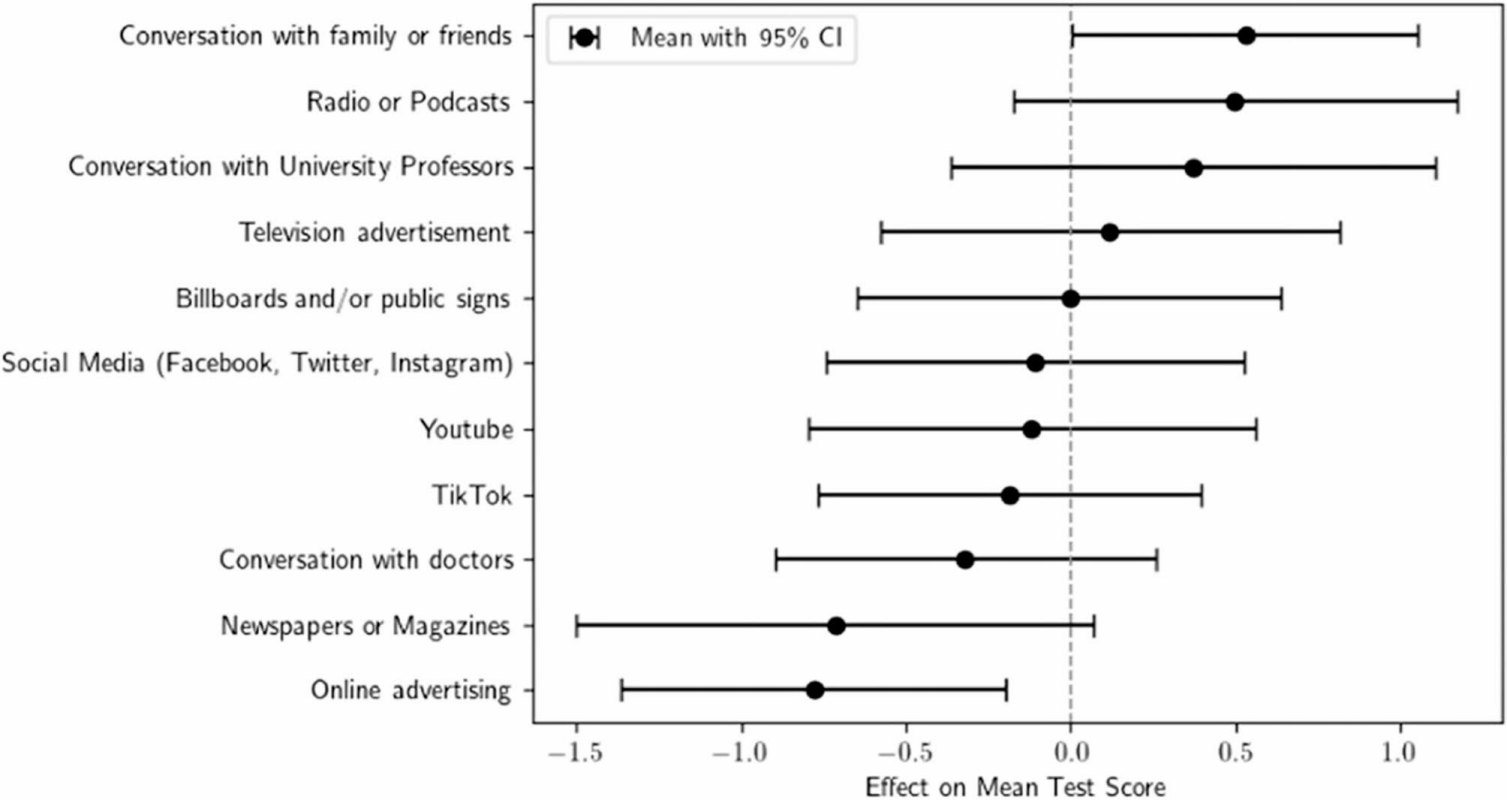
Correlations between sources of information and knowledge scores

There was a significant relationship between students who received their information from online advertising and a lower mean knowledge score (t = -2.672, *p* < 0.01) (Fig. 5).

Among the 80 students surveyed, 79 agreed that vapes are harmful. Fourteen students (17.72%) acknowledged that vaping is a good option for smokers trying to quit, and 19 students (24.05%) thought that vapes are less harmful to health than traditional cigarettes are.

## Discussion

This survey aimed to assess the attitudes, knowledge, and confidence surrounding the use of e-cigarettes among Australian medical students. Unfortunately, the response rate was low, but other surveys were conducted with University of Adelaide medical students. Within our study cohort, 9% of the respondents reported that they were current users of vapes or cigarettes, which is comparable to the percentage of users of tobacco products in the young adult population (18–24 years) in the Australian cohort [30]. However, 41% had previously tried vapes, cigarettes, or shisha. It is important to acknowledge the risk of recruiting a biased audience when a specific group is surveyed, particularly one that may have a vested interest in the topic, which could distort the results.

Notably, a significant portion of the students reported having family members or close friends who were current vape users. This perhaps reflects the growing popularity of vaping within the general public or the fact that perhaps the respondents were an audience invested in the subject at hand.

Interestingly, the majority of the students did not view e-cigarettes as an appropriate alternative for individuals trying to quit traditional cigarettes. This aligns with the position of the NHMRC [10].This belief is also echoed by the TSANZ, which supports the use of e-cigarettes as smoking cessation aids only under the supervision of a healthcare professional and with behavioural support [9].

There was a noticeable tendency towards a polarized view of e-cigarettes among students. When given the option to select a neutral stance in questions about their attitudes, most were more decisive in their positions instead. This may reflect the divisive nature of the topic. In our study cohort, a slight majority (57.5%) of the students believed that e-cigarette use should be banned in Australia, 16.3% disagreed and 26.3% were undecided. There is perhaps scope to consider further defined regulations of e-cigarettes and other novel products following evaluation of the impact of the new legislation.

There was no significant difference in factual knowledge about e-cigarettes across the three clinical years, with an average of 64% correct answers. This finding supports our hypothesis that the year level has a minimal effect on knowledge scores. Ideally, students should gain more knowledge as they progress through medical school due to formal education. This further suggests that there is a significant gap that is not being addressed regarding e-cigarette education.

There was a significant lack of confidence for students across all year groups when discussing the contents of e-cigarettes. This is likely directly related to the large percentage (94%) of students who did not feel that they received adequate education. This, in practice, is concerning when considering that final-year medical students will soon be acting in the role of health providers.

These findings were similar to those from previous surveys, reflecting a significant gap to be addressed in medical school training multiple times nationally.

Interestingly, e-cigarette users were more confident than nonusers but were not necessarily more knowledgeable. This may be because students who use e-cigarettes feel that their personal experience validates their understanding, especially when they discuss the topic with family members or patients.

Although there was no significant difference in knowledge between the male and female medical students, female students reported feeling more confident in discussing e-cigarettes with family and patients. This differs from the findings of other surveys, which revealed that male students were more confident in discussing the topic [22].

There was an association with an increase in confidence among those who had previously tried cigarettes and e-cigarettes, which is similar to the findings of prior studies in different populations of medical students [22].

The study revealed that students primarily obtained information about e-cigarettes from social media rather than from written literature. This finding is similar to those of previous studies in other countries [29]. Given the prevalence of misinformation from unverified online sources, it is concerning—although not surprising—that future medical professionals are relying on these platforms for crucial health information. E-cigarettes are heavily promoted across multiple channels, including online banner and video advertisements, social media, print media, television, and both physical and online retail environments [31]. A small Australian study using a convenience sample reported that, in 2019, more than half (56%) of adult respondents reported exposure to e-cigarette advertisements on social media platforms [32]. Thus, our finding that students who obtained information through online advertising were less knowledgeable about e-cigarettes is particularly intriguing. Although only 11% of the students reported receiving information about vapes from university professors, the significance of university teaching may have been underestimated, as not all teaching staff hold the title of professor. Using more inclusive wording in the survey question may have yielded a more accurate reflection of the sources of information provided by university educators.

This raises questions about the best approaches to educate medical students regarding emerging products such as e-cigarettes. Students indicated a preference for teaching e-cigarettes during the clinical years of medical school. A majority (56%) favoured small group tutorials over lectures and online materials (Fig. 5). This preference may reflect the value students place on interactive formats that allow open discussion and peer engagement. Key topics for such discussions might include the spread of misinformation, the role of social media in shaping perceptions, and the importance of promoting accurate, evidence-based content across communication platforms.

Given that most e-cigarette users are young people, who also represent the largest cohort of social media consumers, these sessions offer a valuable opportunity to contrast social media content with traditional scientific literature, examine the risks of misinformation, and explore the role of medical professionals in guiding patients toward credible sources.

Educational sessions may also include emerging nicotine products, such as nicotine pouches, to ensure that medical students remain informed about the rapidly evolving nicotine landscape. These discussions would likely fall under the broader topic of smoking cessation and can be included alongside pre-existing educations about smoking/cigarettes. Future research could explore effective ways to integrate education on e-cigarettes as a component of smoking cessation training within medical curricula across Australasia and globally.

### Strengths

This study addresses a timely and socially relevant issue, filling a notable gap in current research. Focusing on an underexplored topic provides valuable insights that may inform the development of medical education and curricula. The inclusion of Australian medical students, a population that has been minimally studied in this context, is a key strength, particularly given Australia’s relatively proactive stance on vaping regulation. This focus allows for the exploration of perspectives specific to the Australian context and supports medical educators in advocating for evidence-informed curriculum reforms.

### Limitations

Several limitations must be acknowledged. First, the sample size was relatively small (*n* = 102), and a notable portion of the responses (*n* = 22) were incomplete and excluded from the analysis. This may have introduced bias and limited the generalisability of the findings.

Second, the regulatory environment surrounding e-cigarettes is actively developing. In 2024, e-cigarette use in Australia became pharmacy specific, with ongoing advocacy around restricting access to prescription-only use. These dynamic changes may have influenced participants’ responses, adding complexity to the interpretation of the findings.

Finally, emerging nicotine products such as nicotine pouches and pod models were not widely recognized at the time of data collection. This highlights the speed at which the nicotine market continues to evolve, reinforcing the need for ongoing research and adaptive medical education strategies.

## Conclusion

In conclusion, this investigation of Australian medical students’ knowledge and beliefs about vaping underscores a pressing and critical issue: significant gaps in education that put the health of the public at risk. With misinformation permeating the media and the number of young Australians turning to e-cigarettes on the rise, it is imperative that medical curricula be adapted and strengthened to equip future healthcare providers with comprehensive, accurate, and current knowledge. This is not just an academic exercise but also a public health necessity. Medical students must be prepared not only to understand the complexities of vaping but also to guide patients effectively, offering evidence-based advice that can prevent the potential escalation of vaping-related health problems.

The burden of prescribing e-cigarettes currently falls squarely on the shoulders of these future prescribers, many of whom have demonstrated an inadequate grasp of the subject. Without targeted and proactive educational reforms, we are setting them up for failure and, by extension, jeopardizing the well-being of their patients. Australia’s groundbreaking history of tobacco control and its positive impact on public health provide a powerful model. This serves as a reminder that meaningful change, while challenging, is both achievable and necessary. We must be proactive in addressing these knowledge deficits now to foster a generation of informed, confident healthcare professionals who can navigate the complexities of vaping and support their patients with the clarity and certainty they deserve. The time for this crucial change is not tomorrow; it is today.

## Supplementary Information

Supplementary Material 1.

Supplementary Material 2.

## Data Availability

The datasets used in the study will be available from the corresponding authors upon reasonable request.

## Abbreviations

TGA: Therapeutic Goods Administration
NHMRC: National Health and Medical Research Council
ANU: Australian National University
TSANZ: Thoracic Society of Australia and New Zealand
