# Supplementary material for: Clearing the fog: Australian medical students and the e-cigarette knowledge void – a cross-sectional survey
Source: BMC Med Educ. 2025 Nov 17;25:1612. doi: 10.1186/s12909-025-08126-2 (PMC12625367; doi:10.1186/s12909-025-08126-2)
Supplement: Supplementary file 1 — Supplementary Material 1. [file 12909_2025_8126_MOESM1_ESM.pdf]

Clearing the Fog: Australian Medical Students and the E-Cigarette Knowledge Void – A Cross-Sectional Survey

Information sheet: DO NOT SKIP

1. Please follow the link below for the information page about the survey before completing:

Participant Information Sheet

- ◯ I have read the information sheet above and understand that by completing the survey, I consent to be involved.
- ◯ I have read the information sheet above and DO NOT consent to be involved.

Demographics

2. What is your current year level?

- ◯ Year 4
- ◯ Year 5
- ◯ Year 6

3. What gender do you identify as?

- ◯ Female
- ◯ Male
- ◯ Other
- ◯ Prefer not to say

4. Have you ever tried cigarettes, shisha or vapes?

- ◯ Yes
- ◯ No

5. Do you currently use vapes or tobacco cigarettes?

- ◯ None
- ◯ Tobacco cigarettes only
- ◯ Vapes only
- ◯ Both vapes and tobacco cigarettes

6. Do any of your immediate family or close friends use vapes?

- ◯ Yes
- ◯ No

Knowledge

7. What percentage of Australians aged 13-24years old have ever used vapes?

- ◯ 9%
- ◯ 13%
- ◯ 21%
- ◯ 63%

8. Regarding the legislation surrounding the purchase of vapes in Australia, which of the following is true?

- ◯ Vapes can be purchased at any store or retailer
- ◯ Only vapes containing nicotine require a prescription
- ◯ It is illegal to purchase a vape
- ◯ All vapes require prescriptions

9. Vapes have been found to contain which of the following?

- ☐ Nicotine levels equivalent to 50 cigarettes
- ☐ Acetone (found in nail polish remover)
- ☐ Acrolein (found in weed killers)
- ☐ All of the above
- ☐ None of the above

10. In South Australia, it is illegal to sell vapes to a person under the age of 18.

- ◯ True
- ◯ False

11. Which of the following are known adverse effects of vaping?

- ◯ Vaping has been associated with cancer
- ◯ Vaping has been associated with acute lung disease
- ◯ Vaping has been associated with the development of thyroid disease
- ◯ Vaping has been associated with the development of increased sun sensitivity
- ◯ All of the above

12. Which of the following statements is true about vaping?

- ◯ It is legal to vape in any public place as long as there are no smoke detectors present.
- ◯ Vaping is only illegal in certain indoor public spaces such as schools and hospitals
- ◯ Vaping is illegal in all public places that are designated as smoke-free
- ◯ There are no laws in regardto vaping in public spaces

13. Which of the following statements is true about young people who vape?

- They are less likely to smoke cigarettes than those who don't vape
- They are just as likely to smoke cigarettes as those who don't vape
- They are more likely to smoke cigarettes than those who don't vape

14. The 2023 new health reforms propose pharmaceutical (unbranded) packaging for vapes, as well as restriction on their ingredients and concentration

- True
- False

Attitudes

15. Vaping is harmful to health.

- Agree
- Neither agree nor disagree
- Disagree

16. Vapes are less harmful to health than traditional cigarettes.

- Agree
- Neither agree nor disagree
- Disagree

17. Vapes pose a lower risk of developing cancer than traditional cigarettes.

- Agree
- Neither agree nor disagree
- Disagree

18. Vapes are addictive.

- Agree
- Neither agree nor disagree
- Disagree

19. Vapes should be banned.

- Agree
- Neither agree nor disagree
- Disagree

20. Vaping is better for my patients than smoking tobacco products.

- Agree
- Neither agree nor disagree
- Disagree

21. Vaping is a good option for smokers who are trying to quit smoking cigarettes.

- ◯ Agree
- ◯ Neither agree nor disagree
- ◯ Disagree

22. Helping patients who vape is just like helping smokers or drug users.

- ◯ Agree
- ◯ Neither agree nor disagree
- ◯ Disagree

Confidence and Capabilities

23. I feel confident to discuss the harmful effects of vaping with patients.

- ◯ Agree
- ◯ Neither agree nor disagree
- ◯ Disagree

24. I feel confident to discuss the harmful effects of vaping with patients.

- ◯ Agree
- ◯ Neither agree nor disagree
- ◯ Disagree

25. If a sibling or younger relative were to approach me seeking advice about vaping, I would be confident to discuss its impacts.

- ◯ Agree
- ◯ Neither agree nor disagree
- ◯ Disagree

26. I can confidently discuss the contents of vapes with patients.

- ◯ Agree
- ◯ Neither agree nor disagree
- ◯ Disagree

27. I am confident in my ability to assess and quantify vape use.

- ◯ Agree
- ◯ Neither agree nor disagree
- ◯ Disagree

28. I am confident in recommending treatments for patients who vape.

- ◯ Agree
- ◯ Neither agree nor disagree
- ◯ Disagree

Education

29. Have you received any formal education about vaping in medical school?

- ◯ Agree
- ◯ Neither agree nor disagree
- ◯ Disagree

30. Do you believe that you have received adequate education about vaping in medical school?

- ◯ Agree
- ◯ Neither agree nor disagree
- ◯ Disagree

31. Which part of the medical school curriculum do you believe is best suited to receive education about vaping?

- ◯ Pre-clinical curriculum
- ◯ Clinical curriculum
- ◯ Optional/Elective curriculum

32. What resources would be the most useful to aid your education about the legislation, use and effects of vaping

- ◯ Lectures
- ◯ Small group tutorials
- ◯ Online courses
- ◯ Online resources and guides

33. Have you received any information about vapes outside of medical school? Tick all options that are applicable.

- [ ] Social Media (Facebook, Twitter, Instagram)
- [ ] TikTok
- [ ] Youtube
- [ ] Online advertising
- [ ] Radio or Podcasts
- [ ] Television advertisement
- [ ] Billboards and/or public signs
- [ ] Newspapers or Magazines
- [ ] Conversation with family or friends
- [ ] Conversation with doctors
- [ ] Conversation with University Professors
