# Supplementary material for: Clearing the fog: Australian medical students and the e-cigarette knowledge void – a cross-sectional survey
Source: BMC Med Educ. 2025 Nov 17;25:1612. doi: 10.1186/s12909-025-08126-2 (PMC12625367; doi:10.1186/s12909-025-08126-2)
Supplement: Supplementary file 2 — Supplementary Material 2. [file 12909_2025_8126_MOESM2_ESM.pdf]

Participant Information Sheet

PROJECT TITLE: What are medical students' knowledge and attitudes towards e-cigarettes?

HUMAN RESEARCH ETHICS COMMITTEE APPROVAL NUMBER: H-2023-277

PRINCIPAL INVESTIGATOR: Professor Hubertus Jersmann

STUDENT RESEARCHERS: Samiksha Mali and Vimbiso Chiodze

STUDENT'S DEGREE: Bachelor of Medicine/Bachelor of Surgery

Dear Participant,

You are invited to participate in the research project described below.

What is the project about?

Medical students have a crucial role in shaping society's perspective on vaping as both young individuals and future healthcare professionals. This research project aims to assess medical students' knowledge and their current perspective on e-cigarettes. Evaluating Australian medical students may help us to identify any gaps in understanding regarding e-cigarettes which may direct future teaching. It's important to note that this study's scope is limited to medical students solely from the University of Adelaide, which could potentially impact the wider generalization of the findings.

Who is undertaking the project?

This project is being conducted by Professor Hubertus Jersmann and student researchers; Samiksha Mali and Vimbiso Chiodze, final year medical students at the University of Adelaide. This project is under the supervision of Professor Hubertus Jersmann. There are no external sponsors of the project.

Why am I being invited to participate?

You are being invited to participate in this study as you are a medical student in your fourth, penultimate and final years at the University of Adelaide. Please do not proceed with this questionnaire if you are not within this population.

What am I being invited to do?

Before proceeding, please ensure to read this participant information sheet in full and direct any questions to the researchers.

This online questionnaire consists of 35 multiple-choice questions. Your participation is valued, and we encourage you to provide your best efforts in answering all questions. Some questions are designed to gauge your understanding of e-cigarettes and legislation. Kindly avoid utilizing external resources or seeking assistance from others when addressing these specific questions. Your responses will remain confidential and will not impact your university assessment in any way.

How much time will my involvement in the project take?

The survey is expected to take around 15 minutes to finish, though it's important to note that there is no strict time constraint. Your participation in this study is entirely voluntary, and there won't be any compensation provided for taking part. Please ensure that each participant submits only one response.

THE UNIVERSITY of ADELAIDE

Are there any risks associated with participating in this project?

There are no foreseeable risks that are associated with participating in this project. However, in the event on an incident or adverse event, the appropriate procedure will be followed. We recognize that dedicating time to complete the questionnaire is a commitment, and we have endeavoured to ensure that its length is manageable.

What are the potential benefits of the research project?

The study's outcomes could illuminate the knowledge, perspectives, and convictions of medical students regarding e-cigarettes. This could potentially uncover areas of knowledge deficiency that might shape future adjustments in the medical curriculum. Engaging in this questionnaire could also facilitate participants in introspectively assessing their own knowledge, attitudes, and proficiencies. Such introspection may influence their future interactions with patients and contribute to their broader engagement within the community.

Can I withdraw from the project?

Involvement in this project is entirely voluntary. Should you choose to participate, you retain the right to withdraw from the study at any stage before submitting the survey. Given the assurance of anonymity, any responses submitted cannot be retrieved once provided.

What will happen to my information?

All data collected will be kept anonymous throughout the completion of the study.

The data collected from the questionnaire will be stored in a password protected Excel sheet, accessible only to the lead researchers and supervisors. This data will be stored for 5 years.

The information collected will be analysed and compared with the results of different studies to draw conclusions. Research papers resulting from the analysis of the data will be produced and published in peer reviewed journals and presented at research meetings or poster competitions.

It is important to acknowledge that the data gathered from this study might be utilized in subsequent research endeavors by the research team. By submitting a fully completed questionnaire, you grant consent for the reuse of this data.

Your information will only be used as described in this participant information sheet and it will only be disclosed according to the consent provided, except as required by law.

Who do I contact if I have questions about the project?

If you have any questions or require further information about the study, please do not hesitate to contact the researchers involved. The contact details for all researchers are listed below.

Principal supervisor: Professor Hubertus Jersmann

Email: Hubertus.jermann@adelaide.edu.au

Student researcher: Samiksha Mali

Email: Samiksha.mali@student.adelaide.edu.au

Student researcher: Vimbiso Chiodze

Email: Vimbiso.chiodze@student.adelaide.edu.au

What if I have a complaint or any concerns?

The study has been approved by the Human Research Ethics Committee at the University of Adelaide (approval number H-2023-277). This research project will be conducted according to the NHMRC National Statement on Ethical Conduct in Human Research 2007 (Updated 2018). If you have questions or problems associated with the practical aspects of your participation in the project, or wish to raise a concern or complaint about the project, then you should consult the Principal Investigator. If you wish to speak with an independent person regarding concerns or a complaint, the University's policy on research involving human participants, or your rights as a participant, please contact the Human Research Ethics Committee's Secretariat on:

Phone: +61 8 8313 6028

Email: hrec@adelaide.edu.au

Post: Level 3, Rundle Mall Plaza, 50 Rundle Mall, ADELAIDE SA 5000

Any complaint or concern will be treated in confidence and fully investigated. You will be informed of the outcome.

If I want to participate, what do I do?

To participate, please ensure that you have read the full participant information sheet. By completing the questions and submitting the survey, you acknowledge that you have read and understood what is required and consent to be involved in the study.

Yours sincerely,

Professor Hubertus Jersmann (MD, FRACP)

Samiksha Mali (MBBS VI)

Vimbiso Chiodze (MBBS VI)
